# Great American Smokeout — November 20, 2014

**Published:** 2014-11-14

The Great American Smokeout, sponsored by the American Cancer Society, is an annual event that encourages smokers to make a plan to quit, or to plan in advance and quit smoking on that day, in an effort to stop permanently (1). The 39th annual Great American Smokeout will be held on November 20, 2014.

In the 50 years since the first Surgeon General’s report on smoking and health, cigarette smoking among U.S. adults has been reduced by half. However, more than 20 million persons have died because of smoking, the leading preventable cause of disease, disability, and death in the United States (2).

Nearly two out of three adult smokers want to quit smoking, and more than half had made a quit attempt in the preceding year (2). However, almost one out of five U.S. adults regularly uses one or more combustible tobacco products, such as cigarettes, cigars, pipes, and hookahs (3).

Quitting smoking is beneficial to health at any age and has immediate and long-term benefits. Cutting back rather than quitting completely does not produce significant health benefits. Getting proven, effective help through counseling and medications can increase the chances of quitting successfully two- to three-fold (4).

Additional information and support for quitting is available by telephone (800-QUIT-NOW [800-784-8669]). CDC’s Tips from Former Smokers campaign features real persons living with the consequences of smoking-related diseases and offers additional quit resources at http://www.cdc.gov/tips.

## References

[1] American Cancer Society (2014). Great American Smokeout.

[2] US Department of Health and Human Services (2014). The health consequences of smoking—50 years of progress: a report of the Surgeon General.

[3] Agaku IT, King BA, Husten CG (2014). Tobacco product use among adults—United States, 2012–2013. MMWR Morb Mortal Wkly Rep.

[4] Fiore MC, Jaen CR, Baker TB (2008). Treating tobacco use and dependence: 2008 update. Clinical practice guideline.

